# Th17 cell pathogenicity in autoimmune disease

**DOI:** 10.1038/s12276-025-01535-9

**Published:** 2025-09-01

**Authors:** Eunchong Park, Maria Ciofani

**Affiliations:** 1 Bioscience Immunology, Research and Early Development, Respiratory and Immunology, Biopharmaceuticals R&D, AstraZeneca, Gaithersburg, MD USA; 2 Department of Integrative Immunobiology, Duke University School of Medicine, Durham, NC USA; 3 Center for Advanced Genomic Technologies, Duke University, Durham, NC USA; 4 Department of Molecular Genetics and Microbiology, Duke University School of Medicine, Durham, NC USA

**Keywords:** Autoimmunity, T-helper 17 cells

## Abstract

T helper 17 (Th17) cells have been implicated in numerous inflammatory autoimmune diseases. Clinical benefits from targeting Th17 cell-related cytokines, such as IL-17 and IL-23, highlight how knowledge of Th17 cell development and effector function can be translated into treatments for inflammatory disease. Here we discuss the pathogenic roles of Th17 cells in autoimmune diseases such as multiple sclerosis, inflammatory bowel disease and psoriasis, with emphasis on the cytokines, transcriptional regulators and metabolites that influence Th17 cell differentiation and pathogenicity. Moreover, we address how intestinal environments and physiological responses affect Th17 cells in autoimmune diseases. We also examine current and emerging therapeutic strategies aimed at regulating Th17 cell-driven inflammation to mitigate autoimmune diseases.

## Targeting Th17 cell pathways to treat autoimmune disorders

Autoimmune diseases occur when the immune system mistakenly attacks the body. T helper 17 (Th17) cells, a type of immune cell, play a significant role in these diseases. This article reviews Th17 cells’ involvement in conditions such as multiple sclerosis, inflammatory bowel disease and psoriasis. While Th17 cells help protect against infections, their imbalance can worsen inflammation by releasing harmful substances called cytokines. Researchers explore how Th17 cells develop and the factors influencing their behavior, such as diet and gut bacteria. They also discuss current treatments targeting Th17 cells, such as drugs that block specific cytokines or new therapies involving antibodies and cell-based approaches.　This review highlights that understanding Th17 cell biology can improve treatments for autoimmune diseases. Future research may focus on developing more precise therapies by identifying specific markers and pathways involved in Th17 cell activity.

This summary was initially drafted using artificial intelligence, then revised and fact-checked by the author.

## Introduction

Autoimmune diseases are caused by erroneous immune system activation and targeting of self. Multiple immune cells collaborate in the etiology of autoimmune diseases; among these, T helper 17 (Th17) cells play particularly critical pathogenic roles in driving autoimmune inflammation. This Review provides a comprehensive overview of Th17 cells in the context of autoimmunity focusing on multiple sclerosis (MS), inflammatory bowel disease (IBD) and psoriasis. While Th17 cells play protective roles in antifungal and antimicrobial immunity at mucosal barriers, their dysregulation exacerbates inflammation via secretion of proinflammatory cytokines and recruitment of additional inflammatory cells. To understand the pathogenic development of Th17 cells, we delve into the molecular mechanisms that drive the differentiation of Th17 cells, highlighting the roles of cytokines and transcription factors. We also explore the importance of metabolic pathways that control transcriptional regulation and epigenetic modifications of Th17 cells. Physiological changes and environmental factors such as microbiota composition and dietary molecules also modulate Th17 cell responses, and we discuss how these extrinsic elements contribute to the development of pathogenic Th17 cells in autoimmunity. Lastly, we discuss both current therapeutic strategies aimed at specifically targeting Th17 cells such as biologics targeting IL-17 and IL-23, and emerging therapeutic approaches such as antibody–drug conjugates (ADCs) and cell therapies. Overall, we describe the current state of our understanding of Th17 cell biology that can be exploited for improving treatment approaches for autoimmune diseases.

## Th17 cell biology

### Th17 cells in protective immunity

Th17 cells are a subset of CD4^+^ T cells characterized by the expression of the lineage-defining transcription factor RORγt and IL-17A^1^. Th17 cells can be either protective or pathogenic depending on the types of cytokines and additional transcription factors they express. Homeostatic Th17 cells are enriched at mucosal barrier sites such as the small intestine, colon and lung, and their differentiation in the lamina propria of the small intestine largely depends on intestinal bacteria^2,3^. Th17 cells play protective roles against bacterial and fungal infections. For example, Th17 cells rapidly expand and produce IL-22, IL-17A and IL-17F in response to *Citrobacter rodentium* infection^4,5^.

IL-17A, IL-17F and IL-22 are key cytokine effectors of Th17 cells. The IL-17RA pathway is critical to host defense against bacterial infections at mucosal barriers by recruiting neutrophils to the site of infection and promoting the production of antimicrobial peptides^6^. IL-17A is also important in increasing mucosal IgA levels by promoting polymeric Ig receptor expression^7^. Moreover, IL-17A preserves epithelial barrier integrity upon intestinal damage and inflammation^8^. IL-22 is also important for maintaining mucosal barrier integrity by promoting tissue repair and the production of mucins and antimicrobial peptides^9^. These IL-22-mediated functions protect the host from the dissemination of bacteria^10^.

In addition to bacterial infections, Th17 cell-derived IL-17A and IL-17F contribute to antifungal immunity against *Candida albicans*^11,12^. Importantly, in humans, *C. albicans*-reactive intestinal Th17 cells exhibit cross-reactivity to other fungal pathogens, protecting the host from airborne fungi^13^. Taken together, Th17 cells fortify mucosal immunity against bacteria and fungi.

### Th17 cell pathogenicity in autoimmune diseases

Despite the protective roles of Th17 cells in antibacterial and antifungal immunity, dysregulated Th17 cells cause inappropriate inflammatory responses leading to the development of autoimmune disease.

#### MS and EAE

MS is characterized by autoimmune inflammatory demyelination in the central nervous system (CNS). Th17 cells are critical in the pathogenesis of autoimmune neuroinflammation. Indeed, IL-23-driven pathogenic Th17 cells were initially discovered as disease-inducing cells in the MS model of experimental autoimmune encephalomyelitis (EAE)^14–16^. The requirement for Th17 cells in EAE pathogenesis is further demonstrated by the failure of EAE induction with CD4^+^ T cells deficient in regulators critical to Th17 cell development, such as STAT3^17^ or RORγt^1^. Moreover, IL-17A blockade significantly reduces disease severity and incidence in mice^14^. In accordance with findings in animal models, increased levels of Th17 cells and IL-17A are detected in CNS lesions^18–20^, peripheral blood^21–24^ and cerebrospinal fluid (CSF)^25,26^ of patients with MS. Thus, there is overwhelming evidence supporting Th17 cells as the key pathogenic players driving autoimmune neuroinflammation.

Although the CNS is an immune-privileged site surrounded by the blood–brain barrier (BBB), Th17 cells can penetrate the BBB via various chemokine receptors, adhesion molecules and integrins^27^. In particular, adhesion molecules and integrins such as DICAM, integrin αvβ3 and integrin α3β1 (VLA-3) facilitate Th17 cell CNS infiltration^28–30^. Importantly, among these, VLA-3 expression is highly specific to Th17 cells, and the abundance of VLA-3 ligands in the BBB supports the requirement of VLA-3 in Th17 cell transmigration across the BBB^30^.

The penetration of the BBB is also facilitated by IL-17A through the production of reactive oxygen species (ROS) and matrix metalloproteinases from endothelial cells and fibroblasts^14,31,32^. In addition, IL-17A directly stimulates stromal cells and macrophages to induce the expression of proinflammatory cytokines (for example, IL-1β, IL-6, GM-CSF and TNF)^33,34^. IL-17R-induced chemokine expression from endothelial cells, microglia, astrocytes and resident neuroectodermal cells further attract monocytes, macrophages, neutrophils and lymphocytes into the CNS^14,35,36^. Neutrophils contribute to the disruption of the BBB by producing ROS, matrix metalloproteinases, neutrophil extracellular traps, lipocalin-2, cytokines (for example, IL-1β, IL-6, IL-8 and TNF) and chemokines (for example, CCL2, CCL3 and CCL5)^37^. Moreover, IL-17A exacerbates demyelination by inhibiting the maturation of oligodendrocytes and their survival^38^. Although not required for the development of EAE, IL-22, together with IL-17A, disrupts BBB tight junctions^31,39^. IL-22 can also promote demyelination by increasing the Fas-mediated death of oligodendrocytes and suppressing T_reg_ cells^40^.

GM-CSF is another proinflammatory cytokine secreted by Th17 cells and a hallmark cytokine of pathogenic Th17 cells as GM-CSF-deficient Th17 cells fail to induce EAE^41–43^. Consistently, GM-CSF levels are increased in the CSF and serum of MS patients^44,45^. Although GM-CSF expression is not restricted to Th17 cells^43,46^, CNS-infiltrating Th17 cells are the major source of GM-CSF in EAE^47^. GM-CSF aggravates neuroinflammation by stimulating myeloid cells and microglia in the CNS^41,42,48^. GM-CSF is also required to continuously provide a supply of myeloid cells in the CNS by stimulating the release of Ly6C^hi^ monocyte precursor cells from the bone marrow, which differentiate into dendritic cells and macrophages in the CNS^49^.

Th17 cells are highly plastic, readily losing RORγt and IL-17A expression and converting into IFNγ-producing Th1-like cells, termed ex-Th17 cells, in several autoimmune diseases including EAE, MS, colitis and rheumatoid arthritis (RA)^22,47,50–52^. Phenotypically, ex-Th17 cells display increased expression of proinflammatory cytokines such as IFNγ, TNF and GM-CSF compared with Th17 cells or Th1 cells^50^. Therefore, ex-Th17 cells become a critical source of IFNγ that exacerbates EAE^53^. Recent work identifies a homeostatic, stem-like TCF-1^+^ SLAMF6^+^ IL-17A^+^ Th17 cell population as a reservoir of precursors for the generation of encephalitogenic GM-CSF^+^ IFNγ^+^ CXCR6^+^ ex-Th17 cells during EAE^54–56^, highlighting the dynamic nature of Th17 cell identity and effector transdifferentiation during autoimmunity.

In addition to the release of proinflammatory cytokines, the direct contact of Th17 cells with neurons can induce neuronal damage by elevating neuronal intracellular Ca^2+^ levels^57^. Overall, Th17 cell infiltration into the CNS is a critical event in neuroinflammation as it causes inflammation and demyelination by recruiting other immune cells and disrupting the BBB structure.

#### IBD

IBD, including Crohn’s disease (CD) and ulcerative colitis (UC), is characterized by chronic inflammation and the subsequent tissue damage and fibrosis of the gastrointestinal tract. CD is distinguished by multiple noncontinuous inflammatory patches that can occur in any part of the gastrointestinal tract. However, UC affects the distal region of the large intestine. Although the mechanisms initiating IBD remain unclear, in both CD and UC, Th17 cells are thought to play pathogenic roles given that the mucosa and serum of patients with IBD exhibit elevated levels of Th17 cells and Th17 cell-related cytokines such as IL-17A, IL-17F, IL-21 and IL-22^58–61^. However, blockade of IL-17A or IL-17RA is not only ineffective but also detrimental in patients with IBD, resulting in disease exacerbation^62,63^. Accordingly, colitis models demonstrate the aggravation of colitis by IL-17A neutralization or genetic deletion of *Il17a*^64,65^. This is probably attributable to the protective role of IL-17A in maintaining intestinal barrier integrity^8^. Nevertheless, these findings do not contradict the pathogenic role of Th17 cells in colitis as T cell-transfer colitis models are RORγt-dependent^51,66^.

The dependence of colitis on IL-23, a cytokine driving pathogenic Th17 and ex-Th17 cell development^47,67^, supports the pivotal role of Th17 cells in colitis pathogenesis^68,69^. Indeed, STAT4- and T-bet-dependent IFNγ expression from ex-Th17 cells is critical in colitis induction^51,70^. Consistent with findings from mouse models of colitis, a genome-wide association study (GWAS) revealed a significant association between *IL23R* and IBD, corroborating the physiological importance of IL-23-induced pathways in IBD pathogenesis^71^. These findings collectively reveal that IFNγ expression by IL-23-induced ex-Th17 cells is key to colitis development.

Chronic inflammation in IBD induces intestinal fibrosis, and intestinal strictures of patients with CD display increased levels of IL-17A, which stimulates myofibroblasts to produce collagen^72^. In addition, IL-17A promotes epithelial–mesenchymal transition, which worsens intestinal fibrosis^73^. Th17 cells also produce amphiregulin, which promotes myofibroblast proliferation, motility and collagen expression^74^. Thus, Th17 cells additionally contribute to IBD pathology by aggravating intestinal fibrosis in IBD.

#### Psoriasis

Psoriasis is an autoimmune skin disorder characterized by hyperproliferation of keratinocytes, leading to the formation of thickened, red and scaly plaques on the skin. Th17 cells have been identified as key contributors to the pathogenesis of psoriasis, supported by clinical findings: psoriatic skin lesions contain IL-23-producing dendritic cells and Th17 cells^75–77^, and the serum levels of IL-17A, IL-22, and IL-23 are elevated in patients with psoriasis^78^. Furthermore, GWAS identified *IL17RA* and *IL17RE* as factors increasing risk and disease severity in psoriasis patients^79^. Preclinical disease models also demonstrate the role of IL-17E in inducing keratinocyte proliferation and production of proinflammatory cytokines and chemokines^80^, which further recruit neutrophils to the inflamed skin^81^. The proven clinical efficacy of targeting IL-17A and IL-23 in psoriasis treatment corroborates the critical involvement of the IL-17A–IL23 axes in disease pathogenesis^82^. In line with this, a recent clinical analysis demonstrates that IL-23 blockade-responsive patients are distinguished by reduced Th17 cells, while Th17 cells remain activated in patients with poor response^80^. This also implies that human pathogenic Th17 cells are heterogeneous, including IL-23-dependent and IL-23-independent populations.

Preclinical studies suggest that IL-22 is also required for the progression of cutaneous inflammation^83,84^. GWAS revealed *IL22* as a risk allele for psoriasis^85^, and IL-22 expression is increased in T cells from patients^85,86^. Mechanistically, IL-22 impairs keratinocyte differentiation and increases epidermal thickness^87,88^. However, IL-22-driven pathology is not solely dependent on Th17 cells as IL-22 can be produced by other lymphocytes such as γδ T cells^89^ and Th22 cells^90^. In addition to IL-22, IFNγ levels are highly increased in skin lesions, which promotes Th17 cell development by stimulating myeloid cells to produce IL-1 and IL-23^91^. Although this finding suggests a pathogenic role for Th1 cells in psoriasis, further investigation is required to determine whether bona fide Th1 cells or ex-Th17 cells are the source of IFNγ. Therefore, psoriasis is a heterogeneous disease with differential levels of contribution by Th17 cells and additional crosstalk between Th17 cells and other immune cells.

## Regulators of Th17 cell development

The differentiation and effector programming of Th17 cells are dictated by both cell intrinsic and extrinsic factors, many of which have been elucidated via the use of transgenic and gene deficiency mouse models, as summarized in Table 1. Understanding such cues can direct the development of targeted therapeutic interventions for inflammatory autoimmune disorders in which Th17 cells play a major pathogenic function.

**Table 1:** Transgenic mice used in Th17 cell-mediated autoimmune diseases.

Function	Gene	Mutation type	Disease type	Mutation effect	References
Cytokine	*Csf2*	LOF in T cells	EAE	Protection in EAE	^41,42^
	*Ifng*	LOF in Th17 cells	Colitis	Protection in colitis	^51^
	*Il17a*	LOF	EAE	Reduced EAE severity	^268^
		LOF in CD4^+^ T cells	Colitis	Enhanced colitis severity	^64^
	*Il21*	LOF	EAE	Reduced EAE severity	^101^
	*Il22*	LOF	EAE	No effect on disease	^39^
	*Il23p19*	LOF	EAE	Protection in EAE	^15,16^
			Colitis	Protection in colitis	^68,69^
	*Il23p40*	LOF	EAE	Protection in EAE	^15^
			Colitis	Protection in colitis	^69^
	*Il6*	LOF	EAE	Protection in EAE	^98,102,269–271^
	*Tgfb1*	LOF in T cells	EAE	Reduced EAE severity	^272^
		GOF (*Il2*-driven)		Enhanced EAE severity	^98^
	*Bach2*	LOF	EAE	Enhanced EAE severity	^156^
		GOF		Reduced EAE severity	
TF	*Batf*	LOF	EAE	Protection in EAE	^143^
	*Hif1a*	LOF in T cells	EAE	Reduced EAE severity	^158,192^
	*Irf4*	LOF	EAE	Protection in EAE	^144^
		LOF in CD4^+^ T cells	Colitis	Protection in colitis	^273^
	*Junb*	LOF in T cells	EAE	Protection in EAE	^147,148,274^
		LOF in T cells	Colitis	Protection in colitis	^148^
	*Nfkbiz*	LOF	EAE	Protection in EAE	^152^
	*Rora*	LOF in T cells	EAE	Reduced EAE severity	^150^
	*Rorc*	LOF	EAE	Reduced EAE severity	^1^
		LOF in CD4^+^ T cells	T cell transfer colitis	Protection in colitis	^51,66^
	*Smad2*	LOF in T cells	*C*. *rodentium* infection	Defective Th17 cell induction	^125^
			T cell transfer colitis	Defective Th17 cell induction but enhanced colitis severity	^125^
			EAE	Reduced EAE severity	^126^
			CIA	Reduced CIA severity	^128^
	*Smad3*	LOF in T cells	CIA	Enhanced CIA severity	^128^
	*Stat3*	LOF in T cells	EAU, EAE, autoimmune pneumonitis	Protection in disease	^17,275^
		GOF in T cells	Spontaneous autoimmunity	Spontaneous psoriatic arthritis Spontaneous pulmonary inflammation	^276,277^
	*Stat4*	LOF in Th17 cells	Colitis	Protection in colitis	^51^
	*Tbx21*	LOF in Th17 cells	Colitis	Protection in colitis	^51^
			EAE	Protection in EAE	^157^
	*Tcf7*	LOF in Th17 cells	EAE	Enhanced EAE severity	^155^
		GOF in Th17 cells		Reduced EAE severity	
Receptor	*Ahr*	LOF	EAE	Reduced EAE severity	^227^
	*Gp130*	GOF in T cells	Systemic autoimmunity	Spontaneous autoimmunity	^99^
		LOF in T cells	EAE	Protection in EAE	^100^
	*Ifngr1*	LOF in Th17 cells	Colitis	No change in the severity	^51^
	*Il12rb2*	LOF in Th17 cells	Colitis	No change in the severity	^51^
	*Il1r1*	LOF	EAE	Reduced EAE severity	^113^
		LOF in GM-CSF^+^ cells		Reduced EAE severity	^43^
	*Il23r*	LOF in GM-CSF^+^ cells	EAE	Reduced EAE severity	^43^
	*Tgfbr2*	LOF in T cells	EAE	Reduced EAE severity	^278^
Integrin	*Itga3*	LOF in T cells	EAE	Reduced EAE severity	^30^
Metabolic enzyme	*Got1*	LOF in T cells	EAE	Reduced EAE severity	^201^
microRNA	*Let-7*	GOF	EAE	Protection in EAE	^179^
	*miR-146a*	LOF	EAE	Enhanced EAE severity	^180^
	*miR-181c*	LOF	EAE	Reduced EAE severity	^172^
	*miR-183-96-182*	GOF	EAE	Enhanced EAE severity	^170^
	*miR-21*	LOF	EAE	Protection in EAE	^173^
	*miR-223-3p*	LOF	EAE	Reduced EAE severity	^171^
	*miR-301a*	LOF	EAE	Reduced EAE severity	^174^
		GOF		Enhanced EAE severity	
	*miR-326*	LOF	EAE	Reduced EAE severity	^176^
		GOF		Enhanced EAE severity	
	*miR-384*	LOF	EAE	Protection in EAE	^175^
		GOF		Enhanced EAE severity	

*TF* transcription factor, *LOF* loss of function, *GOF* gain of function, *EAU* experimental autoimmune uveoretinitis, *CIA* collagen-induced arthritis.

### T cell activation

Activation via the T cell receptor (TCR) and costimulatory molecules is a prerequisite for T cell effector polarization. Interestingly, mouse Th17 cell polarization requires relatively strong TCR stimulation^92^, and impaired TCR/mTOR signaling reduces Th17 cell polarization and skews differentiation toward the T_reg_ cell fate^93^. Moreover, suboptimal immunological synapse formation in the absence of VLA-3 results in the downregulation of Th17 cell-specific genes in Th17 cells^30^. Similarly, human Th17 cells require sustained TCR signaling^94^. However, either extreme of high level Akt activation driven by CD28 stimulation or complete inhibition of Akt activation antagonizes human Th17 cell polarization^95^. In addition, ICOS stimulation preferentially enhances Th17 cell differentiation compared with CD28 stimulation^96^, and low level TCR stimulation favors human Th17 polarization when CD28 signaling is present^97^. Thus, it appears that optimal levels of crosstalk between TCR and costimulatory signaling drive Th17 cell fate, a model that warrants further investigation.

### Cytokines

Th17 cell differentiation can be recapitulated in vitro by activating naive CD4^+^ T cells in the presence of IL-6 and TGF-β^1,98^. IL-6 induces Th17 cell polarization via activation of STAT3, and IL-6-deficiency results in a significant reduction of Th17 cells in the intestinal lamina propria^1^. The importance of IL-6 signaling for Th17 cells is further demonstrated by gain-of-function or loss-of-function mutations of the IL-6 receptor subunit gp130 in T cells, which induce spontaneous autoimmunity or protection from EAE, respectively^99,100^.

IL-21 can substitute for IL-6 in STAT3 activation and Th17 cell polarization, and autocrine IL-21 produced by Th17 cells further amplifies proinflammatory Th17 cell responses^101–104^. While IL-6 is likely to play a more dominant role in initiating Th17 cell differentiation given the limited sources of IL-21, the significant reduction of lamina propria Th17 cells in *Il21*^−/−^ mice demonstrates the requirement of both IL-6 and IL-21 in Th17 cell differentiation^101,102^.

TGF-β was initially considered to be an anti-inflammatory cytokine as it promotes T_reg_ cell polarization^105^, but its function is modulated by IL-6 to drive Th17 cell differentiation^1,98^. Indeed, TGF-β overexpression increases encephalitogenic Th17 cells, suggesting its context-dependent role^98^. However, some findings questioned the requirement of TGF-β in Th17 cell differentiation as Th17 cell polarization was driven by IL-6, IL-1β and IL-23, without TGF-β, in the presence of anti-TGF-β antibodies^106,107^. Moreover, Th17 cell differentiation remains inducible from CD4^+^ T cells lacking TGF-β receptor 1 (*Tgfbr1*) or TGF-β2 signaling^106^. However, it is notable that *Tgfbr1* ablation or TGF-β2 signaling deficiency significantly decrease Th17 cell differentiation by approximately 50% and 30%, respectively. Therefore, both TGF-β1 and TGF-β2 signals are likely to contribute to Th17 cell polarization, and their receptors can be stimulated by unknown ligands to compensate for the loss of the other^108^. In addition, TGF-β3 drives pathogenic Th17 cell polarization in combination with IL-6, suggesting the possible compensatory roles of other TGF-β superfamily cytokines in Th17 cell polarization^109^. The requirement of TGF-β signaling in human Th17 cell development is also unclear. In one study, TGF-β reduced Th17 cell polarization of human naive CD4^+^ T cells^110^, whereas, in another, TGF-β favored human Th17 cell development by suppressing the alternative Th1 program^111^. This disparity could be attributable to the balance between TGF-β and other cytokines or the source of naive CD4^+^ T cells: peripheral blood versus umbilical cord blood. While human Th17 cells can be polarized in the presence of anti-TGF-β antibodies^111^, it does not rule out the possible involvement of TGF-β signaling mediated by other ligands that stimulate TGF-β receptors.

IL-23 is not required for in vitro Th17 cell differentiation^1,98^ despite the indispensable role of IL-23 in encephalitogenic Th17 cell development^15,16^. Rather, IL-23 confers pathogenic features to Th17 cells given that Th17 cells cultured in the presence of IL-23 and IL-1β induce severe EAE following adoptive transfer, while Th17 cells cultured in the presence of IL-6 and TGF-β without IL-23 are poor at EAE induction^106,112^. IL-23-mediated Th17 cell pathogenicity is achieved by suppressing IL-10 expression and inducing the expression of GM-CSF and IFNγ^47,48,70,112^. Therefore, it is now accepted that Th17 cells polarized by IL-6 and TGF-β represent homeostatic nonpathogenic Th17 cells, and those polarized in the presence of IL-23 become pathogenic Th17 cells in the context of inflammation. IL-23R signaling also mediates the generation of pathogenic GM-CSF^+^ IFNγ^+^ CXCR6^+^ ex-Th17 cells from homeostatic SLAMF6^+^ Th17 cells during EAE^54^.

In addition to IL-23, IL-1 plays an important role in pathogenic Th17 cell development^113^. IL-1R1 expression is induced on Th17 cells by IL-6, and IL-1 signaling increases IRF4 and RORγt expression to support Th17 cell differentiation^114^. Moreover, IL-1 signaling can inhibit Foxp3 expression, which in turn results in increased Th17 cell polarization or converts T_reg_ cells into Th17 cells^114,115^. IL-1 also promotes the expansion of GM-CSF-producing Th17 cells^116^. Thus, along with IL-23, IL-1 is an important cytokine shaping pathogenic Th17 cell phenotypes.

### Cell intrinsic factors

#### Transcription factors

RORγt is the lineage-defining transcription factor of Th17 cells. Th17 cell differentiation does not occur in the absence of RORγt, and ectopic expression of RORγt induces IL-17A expression in CD4^+^ T cells^1^. Toward Th17 cell-specific transcriptional regulation, RORγt is essential for the expression of small set of key Th17 cell effector genes (for example, *Il17a*/*f*, *Il23r* and *Il22*), but also fine-tunes the expression of a broader set of target genes (for example, *Il10*, *Hif1*, *Egln3*, *Foxo1*, *Il7r*, *Il4ra* and *Il12rb2*)^117^. Despite being a critical lineage specifier, RORγt regulates relatively fewer genes compared with other transcription factors such as BATF, IRF4 and STAT3, requiring other transcription factors that collaborate in specifying the Th17 cell regulatory program^117^.

The Th17-driving role of IL-6 is mainly mediated by activating STAT3, which in turn induces RORγt expression^118,119^. The critical role of the STAT3 pathway in Th17 cell development is supported by early-onset systemic autoimmune manifestations caused by gain-of-function mutations of STAT3^120–122^. IL-6 also fine-tunes TGF-β signaling to inhibit Foxp3 expression and STAT1 activity^123,124^. Despite the importance of IL-6, IL-6 alone does not induce sufficient RORγt expression levels to drive Th17 cell differentiation, requiring additional TGF-β signals^1^. TGF-β promotes the development of Th17 cells through both SMAD-dependent and SMAD-independent pathways^108^.

SMAD2 activated by TGF-β signaling promotes Th17 cell differentiation by enhancing IL-6R expression, STAT3 activation and the activity of RORγt^125,126^. By contrast, SMAD3 plays an opposing role, possibly through interfering with the interaction between SMAD2 and RORγt^126,127^. Consistently, SMAD2 and SMAD3 can interact with STAT3 to respectively activate or repress the transcriptional activity of STAT3 on *Rorc* and *Il17a* genes^128^. However, the roles and mechanisms of SMADs in Th17 cell specification are controversial as both SMAD2 and SMAD3 are also reported to enhance Th17 cell development, not through regulating the expression of RORγt, but rather via suppressing the expression of cytokines such as IL-2, IFNγ and IL-4 that antagonize Th17 cell differentiation^129^. Another contradicting study argues that both SMAD2 and SMAD3 are not required for Th17 cell differentiation^130^. The discrepancies between studies could be attributable to differences in T cell activation methods and TGF-β doses used in vitro and differences in disease models, targeted gene deletion methods in transgenic mice and the involvement of other cells such as T_reg_ cells in vivo.

SMAD4 indirectly suppresses Th17 cell development by interacting with SKI, and its inhibitory function is turned off by TGF-β^131^. Although SMAD4 alone lacks suppressive activity, its interaction with SKI, a transcriptional repressor, enables the suppression of *Rorc* expression. However, upon TGF-β stimulation, SKI is quickly degraded, and SMAD4-SKI-mediated Th17 cell suppression is abrogated. This finding elucidates why SMAD4 was reported to be redundant in Th17 cells in previous studies that used TGF-β for in vitro Th17 cell polarization^132,133^ and identifies an important mechanism by which TGF-β reverses SMAD4-mediated Th17 cell inhibition. Non-SMAD pathways triggered by TGF-β contribute to Th17 cell differentiation via various mechanisms. TGF-β favors Th17 cell differentiation by antagonizing Th1 and Th2 differentiation^134,135^. This idea is supported by the finding that Th17 cell differentiation is induced in CD4^+^ T cells by IL-6 alone when Th1 and Th2 differentiation are impaired by deleting both T-bet and STAT6^136^.

TGF-β signaling can also favor Th17 cell differentiation by negatively regulating transcription factors that otherwise inhibit Th17 cell differentiation. SOCS3, which is transcriptionally induced by the IL-6-STAT3 pathway, downregulates the Th17 cell program by suppressing STAT3 in a negative regulatory feedback mechanism^137,138^. However, TGF-β prolongs the activity of STAT3 by inhibiting SOCS3 expression to ensure Th17-specific transcriptional changes^139^. Eomes, a T-box transcription factor, binds to *Rorc* and *Il17a* loci to suppress their expression, while TGF-β activates the JNK–c-Jun pathway to suppress Eomes expression^140^. Gfi-1, a transcriptional repressor induced by T cell activation, is downregulated by TGF-β to promote Th17 cell differentiation as well as T_reg_ cell differentiation^141^. Thus, an important mechanism by which TGF-β signaling enables Th17 cell differentiation is through inhibiting negative regulators of Th17 cells.

Upon TCR stimulation, BATF, along with Ets1, promotes chromatin looping by recruiting CTCF to initiate lineage-specific gene expression^142^. Both BATF and IRF4 cooperatively function as pioneer transcription factors to open chromatin regions to which subsequent transcription factors are recruited^117^. Moreover, both BATF and IRF4 are essential for the induction of RORγt expression^143–145^. Importantly, the overexpression of RORγt is not sufficient to restore IL-17A expression in BATF-deficient CD4^+^ T cells, providing evidence of essential regulation upstream of RORγt expression^143^.

STAT3 is further induced by the cooperative action of BATF and IRF4^117,119^. Positive feedback loops among BATF4, IRF4 and STAT3 amplify their own expression, and they together induce the expression of c-Maf and RORγt. Consistent with the requirement for c-Maf in homeostatic Th17 cell differentiation^146^, c-Maf negatively regulates the expression of proinflammatory genes (for example, *Rora*, *Runx1*, *Il1r1*, *Ccr6* and *Tnf*) and counteracts BATF through a negative feedback loop^117^.

Some AP-1 transcription factors play critical roles in Th17 cell development and the maintenance of Th17 cell identity. While Fosl2 negatively affects IL-17A expression, its loss dysregulates Th17 cell identity allowing the expression of Foxp3, T-bet and IFNγ^117^. Another AP-1 factor that regulates plasticity is JunB, which is required for pathogenic Th17 cell development and maintenance^147^. Due to the context-dependent role of JunB, homeostatic Th17 cell development in the lamina propria is not impaired in the absence of JunB^147,148^. JunB shapes Th17 cell-specific identity by suppressing key genes of alternative Th cell programs (for example, *Foxp3*, *Tbx21* and *Ifng*), and the loss of JunB permits the expression of IFNγ and Foxp3 in Th17 cells^147^. The altered transcriptional program in the absence of JunB can be attributed to IRF8—which replaces IRF4—and JunD—which replaces JunB—in AP-1 complexes^147^.

RORα is another member of the RAR-related orphan receptor family that promotes Th17 cell differentiation^149–151^. Although RORα has fewer target genes than RORγt, it plays nonredundant roles in Th17 cell differentiation. Indeed, the loss of RORα or pharmacological inhibition of RORα downregulates the pathogenicity of Th17 cells in EAE^150,151^. Additional transcription factors, such as Runx1 and IκBζ, work in cooperation with RORγt and RORα as positive regulators of Th17 cell differentiation^152,153^.

The conversion of Th17 cells to pathogenic ex-Th17 cells is regulated by various additional transcription factors. Blimp-1 expression induced by IL-23 promotes proinflammatory gene expression together with RORγt and STAT3^154^. IL-23 also drives generation of pathogenic Th17 cells by suppressing the expression of TCF1 (encoded by *Tcf7*); TCF1 otherwise maintains the homeostatic Th17 cell state by supporting stem-like and regulatory features while repressing inflammatory programs by binding and restraining RORγt activity^155^. Similarly, BACH2 promotes an immunomodulatory Th17 cell state, preventing Th17 cells from acquiring a pathogenic Th1-like epigenetic landscape and cell fate program^156^. Thus, loss of either TCF1 or BACH2 enhances the encephalitogenic capacity of Th17 cells^155,156^. Moreover, GWAS-identified variants are associated with altered *BACH2* expression levels in autoimmunity, further corroborating the critical role of BACH2 in regulating Th17 cell pathogenicity^156^. In addition to the downregulation of negative regulators, the acquisition of IFNγ expression in pathogenic ex-Th17 cells is mediated by the upregulation of T-bet, Runx1 and Runx3, and the ablation of these factors in Th17 cells protects mice from colitis^51^ and EAE^157^. Another proinflammatory cytokine IL-1 enhances the expression of BATF and IκBζ and suppresses Foxp3 expression to further facilitate Th17 cell specification^115^.

In summary, Th17 cell differentiation requires various transcription factors that reinforce Th17 cell-specific regulatory networks. This process first requires TCR stimulation which activates pioneer transcription factors, BATF and IRF4, to regulate specific chromatin accessibility to facilitate subsequent transcription factor recruitment. Together with pioneer factors, in the presence of Th17 cell-driving cytokine signals, STAT3 induces other transcription factors, including RORγt and RORα, that further skew Th17 cell differentiation. While many transcription factors activate gene expression, some transcription factors such as c-Maf and Fosl2 mainly function as negative regulators^117^. Indeed, suppression is as important as activation of gene expression programs as downregulation of the gatekeepers of Th17 lineage identity results in acquisition of alternative lineage fates (for example, JunB) and unleashes the proinflammatory program, making Th17 cells pathogenic (for example, TCF1 and BACH2).

#### Epigenetic and *cis* regulation

Th17 cell differentiation and pathogenicity is controlled by various epigenetic mechanisms, including histone modifications that influence chromatin accessibility and shape lineage-specific gene expression. The global epigenetic landscape of Th17 cells is established by pioneer factors IRF4 and BATF that initiate chromatin accessibility, and together with STAT3, recruit the histone acetyltransferase p300 to Th17 cell enhancers^117^. RORγt mediates histone acetylation at key effector loci, such as *Il17a* and *Il23r*, via interactions with HIF1α and p300^117,158^. In addition, class II histone deacetylases HDAC4 and HDAC7 cooperatively regulate effector gene expression for Th17 cell differentiation through interaction with JunB and Aiolos, respectively^159^. Pharmacological inhibition or genetic deletion of *Hdac4* or *Hdac7* mitigates Th17 cell-mediated inflammation and colitis in mice^159^.

The action of histone-modifying enzymes results in the enrichment of active histone marks such as H3 acetylation and H3K4 trimethylation (me3) in the promoter regions of *Il17a*/*f* and *Rorc* loci in Th17 cells^160,161^. Moreover, repressive H3K27me3 is lost at these type 3 loci, with JMJD3 required for Th17-specific H3K27 demethylation^162^. Notably, Th17 cell effector flexibility has been attributed to the semi-permissive bivalent H3K4me3 and H3K27me3 modification status at the type 1 lineage-defining *Tbx21* (encodes T-bet) locus^161^. Moreover, histones at key cytokine loci (for example, *Il17a*/*f* and *Ifng*) undergo dynamic remodeling during type 1 conversion, revealing an epigenetic instability that facilitates Th17 cell effector plasticity^163^. DNA methylation also impacts Th17 cell identity as CpG demethylation of *Il17a*/*f* promoter establishes an open chromatin status, enabling the binding of STAT3^164^.

At the core of Th17 cell identity are the *cis* elements that control expression of lineage-relevant loci. Conserved noncoding sequence (CNS) 6 and CNS9 are *Rorc* locus enhancers that recruit transcription factors such as STAT3 and c-Maf to induce *Rorc* expression downstream of IL-6 or TGF-β stimulation^165^. The upstream RORCE2 enhancer regulates *Rorc(t)* promoter activity during Th17 cell differentiation via SOX-5-mediated chromatin looping and STAT3 recruitment^166^, while an RORα-dependent + 11 kb enhancer is required for maintenance of RORγt expression^151^. At the *Il17a*/*f* locus, eight CNSs (CNS1–CNS8) are marked with H3 histone acetylation^160^. In particular, CNS2 is an essential enhancer that controls chromatin accessibility at the *Il17a*/*f* locus by recruiting p300 and JMJD3^167^. Moreover, numerous transcription factors, including RORγt, JunB, BATF, I_K_Bζ and Runx1 that are required for Th17 cell differentiation, bind CNS2 and regulate *Il17a* expression^117,143,147,152,153^. Taken together, Th17 cell identity and plasticity is regulated by the concerted action of DNA *cis* elements, epigenetic chromatin status and environment-sensing *trans*-acting transcription factors.

#### Noncoding RNAs

Noncoding RNAs, including microRNAs (miRNAs) and long noncoding RNAs (lncRNAs), have emerged as critical regulators of Th cell polarization^168^. miRNAs function in posttranscriptional gene regulation, reducing target gene expression via mRNA translational suppression or degradation. A network of miRNAs impact various pathways to play either positive or negative roles in Th17 cells^169^. Indeed, miRNAs can promote differentiation by targeting negative regulators of Th17 cells. In particular, the miR-183-96-182 cluster and miR-223-3p enhance Th17 cell pathogenicity by targeting *Foxo1* and *Foxo3*, respectively^170,171^. miR-21 and miR-181c target *Smad7*, whereas miR-384 and miR-301a target *Socs3* and *Pias3*, respectively, to promote Th17 cell polarization^172–175^. Similarly, miR-326 targets Ets-1, a negative regulator of Th17 cells, to support Th17 cell development^176^.

miRNAs also counteract Th17 cell differentiation. miR-29a-3p and miR-21-5p delivered by exosomes can suppress STAT3 in Th17 cells and regulate the Treg/Th17 cell balance^177^. miR-18a inhibits Th17 cells by targeting *Smad4*, *Hif1a* and *Rora*^178^. Some miRNAs impact cytokines or receptors to regulate Th17 cells. Let-7 miRNA is a negative regulator of *Il1r1*, *Il23r*, *Ccr2* and *Ccr5*^179^, while miR-146a inhibits the autocrine production of IL-6 and IL-21 in encephalitogenic T cells, reducing Th17 cell differentiation^180^.

lncRNAs influence gene expression by modulating chromatin structure and function and via posttranscriptional regulation of RNA. In Th17 cells, lncRNA Neat1 enhances *Il17* and *Il23r* expression, and its knockdown protects mice from developing EAU^181^. lncRNA IFNG-AS1 is highly expressed in autoimmune patients and promotes *Ifng* expression in human and mouse CD4^+^ T cells^182–184^; however, it is unclear whether it is active in both ex-Th17 cells and bona fide Th1 cells. lncRNA STAT4-AS1 inhibits Th17 cells by interfering with the interaction between RORγt and *Il17a* promoter^185^. Lastly, lncRNA H19 overexpression inhibits Th17 cell differentiation, potentially by downregulating miR-342-3p^186^. Collectively, noncoding RNAs are critical regulators of Th17 cell biology. Future research focusing on the complex interplay between noncoding RNAs and other regulatory mechanisms will enhance our understanding of the networks controlling Th17 cell pathogenicity.

### Metabolism

The availability of nutrients is a critical determinant of Th cell polarization as Th cell subsets have distinct metabolic requirements^187^. For example, the metabolism of Th17 cells is characterized by aerobic glycolysis, which requires high levels glucose, while T_reg_ cells preferentially utilize lipids to fuel fatty acid oxidation and oxidative phosphorylation^188–190^. However, this does not preclude the requirement for lipids for Th17 cell development. In fact, Th17 cells greatly depend on de novo fatty acid synthesis, whereas T_reg_ cells readily import exogenous fatty acids^191^. Accordingly, the distinctive inherent metabolism of Th17 cells and T_reg_ cells has important consequences when nutrients are restrained; limiting glycolysis reduces Th17 cell development, and oxidative phosphorylation inhibition suppresses T_reg_ cell development^189,192,193^.

The glycolysis-skewed metabolic signature of Th17 cells is established by mTOR and HIF-1α^192^. In Th17 cells, HIF-1α is highly expressed to reinforce glycolysis, and mTOR activation is required for HIF-1α induction. Notably, HIF-1α deficiency results in reduced Th17 cell differentiation and enhanced T_reg_ cell differentiation. In addition to roles in regulating cell metabolism, HIF-1α directly promotes the transcription of *Rorc* and *Il17a* to further Th17 cell development^158^. Moreover, HIF-1α plays a negative role in T_reg_ cells by mediating Foxp3 protein degradation^158^. To support the high demand for glucose, HIF-1α induces the expression of glucose transporter 1 (Glut1) in Th17 cells^192^. Interestingly, in activated CD4^+^ T cells, HIF-1α and downstream Glut1 expression can be regulated by leptin, an adipokine decreased in malnutrition^194,195^. Therefore, leptin signaling enhances glucose uptake in activated CD4^+^ T cells by increasing Glut1 expression in a HIF-1α-dependent manner, thereby promoting Th17 cell differentiation, whereas the reduction of leptin signaling attenuates EAE^194–196^. These findings demonstrate that nutritional status and altered metabolism can regulate Th17 cell development and pathogenicity.

Retinoic acid is well known to regulate the balance between Th17 and T_reg_ cells by reducing Th17 cell polarization while enhancing T_reg_ cell polarization, and it can ameliorate the severity of EAE^197–199^. However, retinoic acid cannot be applied therapeutically to treat autoimmune diseases as it causes hypercalcemia^200^.

A recent study revealed that the unique metabolic program in Th17 cells goes beyond functioning as an energy source^201^. In particular, various metabolic intermediates and byproducts can affect epigenetic modifications^202^. Th17 cells have high levels of 2-hydroxyglutarate (2-HG), a transamination product of α-ketoglutarate (α-KG), and 2-HG causes hypermethylation of the *Foxp3* locus, silencing transcription^201^. Inhibition of the glutamate pathway reduces the level of α-KG and 2-HG, consequently increasing Foxp3 expression. In line with this finding, glutamine metabolism which produces α-KG is required for building a Th17 cell-specific epigenetic landscape and inhibiting the Th1 program^203^. Moreover, methionine-driven *S*-adenosyl-L-methionine biosynthesis is required to drive H3K4 histone methylation at promoter regions of key Th17 genes (for example, *Il17a*, *Il17f* and *Batf*), and methionine restriction downregulates the pathogenicity of Th17 cells in EAE^204^. Because other metabolic intermediates (for example, acetyl-CoA, succinyl-CoA, α-KG, fumarate and palmitic acids) also play roles in epigenetic changes, it is tempting to further investigate possible roles of such metabolites in regulating chromatin accessibility and identity in Th17 cells.

### Other factors

In addition to cytokines, Th17 cells encounter various environmental factors that modulate cellular signals and contribute to Th17 cell polarization and pathogenicity.

#### Gut environment

Th17 cells are enriched in the lamina propria of the small intestine where they encounter various commensal or virulent bacteria. Notably, the microbiota influences Th17 cell development. Indeed, Th17 cells are significantly reduced in the small intestine lamina propria of germ-free mice compared with specific-pathogen-free control mice^2^. In mice, segmented filamentous bacteria (SFB) are sufficient to drive Th17 cell polarization in the small intestine lamina propria^3^. Moreover, SFB colonization provides the host with protection against *C. rodentium*. In addition to homeostatic Th17 cell development, pathogenic Th17 cell development in the context of autoimmunity also depends on gut-resident microbiota. For example, SFB can exacerbate various autoimmune diseases by upregulating Th17 cell responses^205–207^. In germ-free mice or mice with altered gut microbiota, EAE development is delayed or attenuated, demonstrating the essential role of commensal bacteria in pathogenic Th17 cell development^208–210^. In particular, UvrA, a bacterial antigen expressed in *Lactobacillus reuteri*, cross-stimulates MOG-reactive T cells, and *L. reuteri* colonization alone can aggravate EAE in germ-free mice^208^. In addition, surface layer protein A expressed in *Clostridioides difficile*—a bacterium that causes inflammation in the large intestine—displays molecular similarity with MBP, stimulating MBP-reactive T cells^211^. Therefore, molecular mimicry by commensal or virulent bacteria can stimulate pathogenic Th17 cells in autoimmune diseases. In fact, several studies reported altered gut microbiota in patients with MS, reinforcing the idea that intestinal microorganisms are an important pathogenic determinant in autoimmune diseases^212–215^. While commensal bacteria can be either protective or pathogenic depending on the context, these studies collectively demonstrate the critical contribution of gut-residing bacteria to Th17 cell development.

In the case of SFB colonization, the Th17-driving effect of bacteria is attributable to bacterial antigens recognized by T cells, yielding SFB-reactive Th17 cells^216,217^. In addition, the adhesive property of bacteria also induces Th17 cell differentiation as adhesion of bacteria to intestinal epithelial cells promotes the release of serum amyloid A (SAA) and ROS, which in turn stimulates dendritic cells to produce IL-1β^218^.

SAA proteins produced by the small intestine directly affects Th17 cell differentiation^219^. SAA1 can substitute for TGF-β, and Th17 cells cultured with IL-6 and SAA1 display pathogenic phenotypes. Moreover, both systemic and local expression of SAAs promotes pathogenic Th17 cell responses in colitis models and EAE.

Bile acids are primarily synthesized in the liver and secreted into the duodenum, and bacteria in the colon produce secondary bile acids as bacterial metabolites. Derivatives of lithocholic acids (LCAs), secondary bile acids, were recently reported to affect Th17 cell differentiation^220,221^. In particular, 3-oxoLCA and IsoLCA attenuate Th17 cell differentiation by directly inhibiting RORγt activity, and isoalloLCA favors T_reg_ cell differentiation while suppressing Th17 cell differentiation by producing mitochondrial ROS, which increases Foxp3 expression. Moreover, the levels of 3-oxoLCA and isoalloLCA are decreased in patients with CD, suggesting a new mechanism by which bacteria-derived bile acids affect Th17 cell pathogenicity^221^.

Short-chain fatty acids are another type of bacterial metabolite. Although their effects are not restricted to Th17 cells, butyrate and propionate can promote T_reg_ cell development by inhibiting histone deacetylases, which increases acetylation of the *Foxp3* locus and Foxp3 protein^222^. Moreover, butyrate can suppress human lamina propria CD4^+^ T cell activation and proliferation, resulting in reduced production of IL-17A, IL-22 and IFNγ^223^. However, the detailed epigenetic changes induced by butyrate remain unknown. In a conflicting report, short-chain fatty acids acetate, propionate and butyrate promote Th17 and Th1 cell differentiation in vitro, and propionate treatment enhances Th1 and Th17 cell responses upon *C*. *rodentium* infection^224^. Additional investigation is necessary to resolve these contradicting findings that could be attributed to different assay contexts and Th cell polarizing conditions used in these studies.

Aryl hydrocarbon receptor (AHR) is a transcription factor that can recognize various ligands ranging from natural compounds to synthetic ligands^225^. Many naturally occurring AHR ligands such as tryptophan, curcumin and carotenoids can be obtained from plants, and commensal bacteria in the gut can metabolize tryptophan to produce AHR ligands such as indole acetic acid and indole-3-acetaldehyde^226^. Th17 cells express high levels of AHR, and AHR activation promotes Th17 cell differentiation both in vitro and in vivo, increasing the susceptibility to EAE^227,228^. Therefore, an AHR ligand-rich environment is another important environmental factor that favors Th17 cell development in the gut. Moreover, because Iscove’s modified Dulbecco’s medium is enriched with AHR agonists, in vitro polarization and expansion of Th17 cells are more efficient when cells are cultured in Iscove’s modified Dulbecco’s medium versus RPMI 1640 medium^229^.

#### Physiological responses

There are physiological responses required to maintain homeostasis. This includes maintenance of core body temperature. Recent research reveals that inflammation-driven fever can enhance Th17 cell differentiation^230^. Febrile temperature increases the transcriptional activity of SMAD4 via SUMOylation, which further induces the expression of key Th17 cell genes such as *Il17a*, *Il17f*, *Il21*, *Il1r1* and *Il23r*. This finding is seemingly at odds with previous studies reporting the redundancy of SMAD4 in Th17 cell differentiation, which were conducted under normal non-febrile conditions^132,133^. However, under a febrile temperature that mimics a physiological setting for inflammation, SMAD4 functions as a positive regulator of Th17 cell differentiation, suggesting that the role of SMAD4 is temperature-dependent^230^. This finding reveals an additional mechanism by which Th17 cell polarization is influenced by inflammatory conditions in vivo.

Another important homeostatic mechanism occurring in our body is osmoregulation, including sodium homeostasis. Notably, the level of sodium in the extracellular fluid is prone to elevation by a high-salt diet, and high salt levels can promote Th17 cell development and exacerbate EAE^231,232^. Increased NaCl concentration induces serum glucocorticoid kinase 1 expression, leading to the upregulation of IL-23R expression^232^. Similarly, the proinflammatory role of a high-salt diet can aggravate colitis by promoting IL-17 expression in Th17 cells and type 3 innate lymphoid cells^233^.

Many physiological behaviors and events at the molecular level are regulated by homeostatic circadian rhythms, and accumulating evidence suggests a link between the circadian clock and immune responses^234^. Two main transcription factors that govern diurnal gene expression patterns are CLOCK and BMAL1, and their transcriptional activities regulate rhythmic expression of their target genes^235^. The expression of *Rorc* is indirectly regulated by these factors, peaking during the day and revealing how Th17 cell development is linked to the circadian clock^236^. In addition, seasonal changes can regulate physiological processes. Indeed, the immune system displays a circannual pattern in the expression of proinflammatory genes^237^. One of the factors involved in circannual rhythm is melatonin, which is elevated in winter owing to a shorter daytime^238^. Several studies found that the MS relapse rate is variable, showing a seasonal pattern, high in spring and summer^239,240^. Mechanistically, melatonin functions as a negative regulator of Th17 cells by activating NFIL3-mediated *Rorc* inhibition, and seasonally fluctuating melatonin levels show an inverse correlation with MS relapses^241^.

## Therapeutic approaches to target Th17 cell-driven pathogenesis

**Fig. 1:**
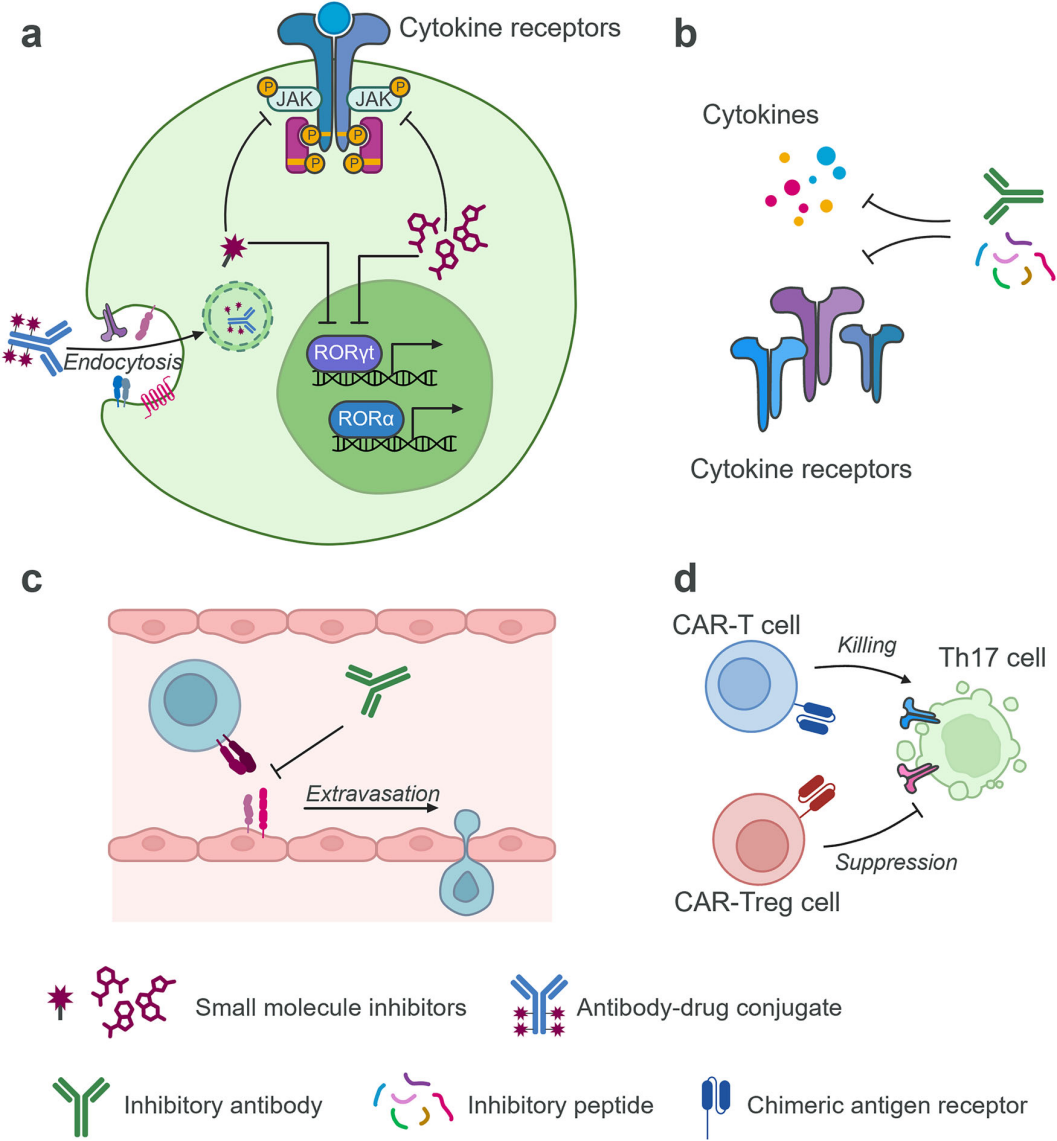
Therapeutic approaches targeting Th17 cell-driven inflammation. **a** SMIs can target JAK isoforms or transcription factors such as RORγt and RORα to suppress Th17 cells. ADC can promote the target-specific delivery of SMIs. **b** Blockade of cytokines or cytokine receptors using antibodies or peptides can inhibit Th17 cell-mediated inflammation. **c** Inhibitory antibodies targeting integrins or adhesion molecules can inhibit the infiltration of Th17 cells into the site of inflammation. **d** CAR-T cells can directly kill pathogenic cells in Th17 cell-driven inflammation. CAR-T_reg_ cells can suppress inflammation driven by Th17 cells.

Given the pathogenic roles of Th17 cells in various autoimmune diseases, Th17 cells are a promising target for therapeutics. Current trials are using diverse modalities to directly or indirectly target Th17 cell biology (Fig. 1).

### Small-molecule inhibitors

The advantage of small-molecule inhibitors (SMIs) is their ability to target intracellular signaling molecules and transcription factors. Notably, blocking the JAK–STAT pathway is effective at suppressing proinflammatory cytokine signaling pathways responsible for autoimmune disease (Fig. 1a). Several inhibitors targeting each of the four JAK isoforms (JAK1, JAK2, JAK3 and TYK2) are currently Food and Drug Administration-approved or under clinical trials to treat various autoimmune diseases, but their effects are not necessarily restricted to Th17 cells^242^.

For specific targeting of Th17 cells in autoimmune disease, RORγt has been suggested as an ideal therapeutic target, and many clinical trials have tested the efficacy of RORγt antagonists in psoriasis^243^ (Fig. 1a). However, most RORγt inhibitors show poor safety or poor efficacy. A major concern of RORγt inhibition is thymocyte apoptosis^244^. Alternatively, a preclinical study demonstrates the therapeutic benefit of an RORα antagonist in treating EAE without affecting thymic T cell development^150^ (Fig. 1a). However, the clinical benefits of RORα inhibition are yet to be tested.

ADCs are believed to increase the efficacy and specificity while decreasing the toxicity of SMIs. Although current ADC development is mainly focused on cancer treatment, it can be applied to inflammatory disease as demonstrated by IL-7R-ADC, which attenuates arthritis in a mouse model by targeting IL-7R-expressing inflammatory T cells^245^. Given the critical pathogenic functions of Th17 cells in autoimmune diseases such as RA^246^ and psoriasis^247^, it is tempting to develop ADC to suppress Th17 cell-driven inflammation. Further identification of pathogenic Th17 cell-specific surface markers can help to appropriately target ADCs to proinflammatory Th17 cells (Fig. 1a).

### Antibodies: cytokine blockade

Blocking cytokines can counteract the pathogenic function of Th17 cells (Fig. 1b). Indeed, targeting the IL-17A pathway via the IL-17A inhibitor secukinumab or the IL-17RA inhibitor brodalumab is clinically efficacious in the treatment of psoriasis^82^. By contrast, anti-IL-17A therapy has shown no or limited clinical benefits in other autoimmune diseases such as MS or RA^248^, and one possible explanation is SHP2-mediated IL-17-independent IL-17R signaling that nullifies the blockade of IL-17A^249^. In the case of IBD, IL-17A blockade worsens disease^62,63^, probably due to the pleiotropic role of IL-17A in maintaining intestinal integrity^8,64^.

IL-23-blocking antibodies such as guselkumab, risankizumab and tildrakizumab or IL-12/IL-23-blocking antibody ustekinumab demonstrate therapeutic efficacies in several autoimmune diseases, including IBD and psoriasis^82^. As an alternative to antibody-based inhibitors, an oral peptide blocking IL-23R demonstrates therapeutic benefits in psoriasis^250^ and in a preclinical model of IBD^251^. However, the resistance of some Th17 cell populations to IL-23 blockade in psoriasis suggests the presence of alternative pathways that support pathogenic Th17 cells^80^. Compared with psoriasis, hidradenitis suppurativa exhibits distinct Th17 cell populations that are more dependent on IL-1β than IL-23^252^. Such Th17 cell heterogeneity within and across different inflammatory diseases results in varying levels of clinical response to treatment and presents a challenge in developing effective therapeutics.

IL-6 is also targetable given its role in driving Th17 cell development, and tocilizumab, an anti-IL-6R antibody, is approved to treat patients with RA. However, tocilizumab induces psoriasis-like symptoms in patients with RA^253,254^ and worsens symptoms in some patients with psoriasis^255^. One possible explanation for the adverse effect of anti-IL-6 therapies in skin inflammation is the compensatory increase of other proinflammatory cytokines by keratinocytes upon IL-6 blockade^256^. In the treatment of RA and psoriasis, IL-22/IL-22R antagonists fail to show clinical benefits^257^. Lessons learned from failed clinical trials indicate that (1) knowledge obtained from preclinical models is not always transferrable to human conditions, and (2) a better understanding of the complex interplay between cytokines in each autoimmune disease as well as disease heterogeneity is necessary to develop more effective and precise anti-cytokine therapy.

### Blocking tissue infiltration

The blockade of lymphocyte migration into inflamed tissues is achievable by an anti-integrin α4 (natalizumab) or S1P receptor inhibitors, which are currently used to treat patients with MS and those with IBD^258,259^. However, these therapies are not effective in inhibiting Th17 cells^260–262^, necessitating the identification of Th17 cell-specific migration mechanisms. Recent preclinical studies identified several surface proteins facilitating Th17 cell CNS migration such as integrin αvβ3^29^, DICAM^28^ and VLA-3^30^. While clinical benefits of targeting these newly identified molecules are yet to be tested, inhibiting Th17 cell infiltration into the site of inflammation has high potential in ameliorating Th17-mediated autoimmune disorders (Fig. 1c).

### Cell therapies: chimeric receptors

Chimeric antigen receptor (CAR)-expressing cytotoxic T cells can directly recognize and kill target antigen-expressing cells. Several clinical trials using CAR-T cell therapies targeting CD19 or BCMA are underway to assess their efficacy in eradicating autoantibody-producing B cells in autoimmune diseases^263,264^. Moreover, the use of chimeric autoantibody receptor enables the removal of autoantigen-specific B cells^265^. In addition to B cell depletion, the elimination of autoreactive T cells is probably achievable by developing peptide-HLA-chimeric-receptor-expressing T cells^266^. The recent advances in cell therapy using chimeric receptors provide an impetus to develop novel CAR-T cells that either directly kill pathogenic Th17 cells or target other cells that promote Th17 cell pathogenicity (Fig. 1d). In an alternative approach, IL-23R-CAR T_reg_ cells are being developed to suppress inflammation driven by the IL-23–Th17 axis in patients with IBD^267^ (Fig. 1d). Investigation into additional Th17 cell-selective markers will enable precise targeting of Th17 cell-mediated pathogenic pathways in autoimmunity.

## Conclusion

Since their discovery, the pathogenic role of Th17 cells in various autoimmune diseases has been at the center of research efforts. The pathogenic features of Th17 cells are imparted by numerous factors, including cytokines, transcription factor regulation and metabolism. The identification of such factors is critical in therapeutic targeting of Th17 cells as exemplified by IL-23/IL23R-targeting therapies in IBD and psoriasis. However, several clinical trials have revealed discrepancies in therapy effectiveness across inflammatory diseases, which can be attributable to the heterogeneity of Th17 cells and tissue-specific contexts. Therefore, deconvoluting this heterogeneity will help to identify therapeutic targets in specific patient groups. While classical drug development has relied on the use of SMIs, recent advances in our knowledge and genetic engineering technologies have diversified treatment modalities including cell therapies and antibody-based biologics such as ADC. Further identification of Th17 cell markers and regulatory factors will aid in developing strategies to treat autoimmune inflammatory diseases in which Th17 cells play a major pathogenic role.
